# Exploring public sentiment and vaccination uptake of COVID-19 vaccines in England: a spatiotemporal and sociodemographic analysis of Twitter data

**DOI:** 10.3389/fpubh.2023.1193750

**Published:** 2023-08-17

**Authors:** Tao Cheng, Baoyan Han, Yunzhe Liu

**Affiliations:** SpaceTimeLab, University College London, Civil, Environmental and Geomatic Engineering, London, United Kingdom

**Keywords:** COVID-19, vaccine, social media, sentiment analysis, vaccination intention, sociodemographic analysis, spatiotemporal analysis

## Abstract

**Objectives:**

Vaccination is widely regarded as the paramount approach for safeguarding individuals against the repercussions of COVID-19. Nonetheless, concerns surrounding the efficacy and potential adverse effects of these vaccines have become prevalent among the public. To date, there has been a paucity of research investigating public perceptions and the adoption of COVID-19 vaccines. Therefore, the present study endeavours to address this lacuna by undertaking a spatiotemporal analysis of sentiments towards vaccination and its uptake in England at the local authority level, while concurrently examining the sociodemographic attributes at the national level.

**Methods:**

A sentiment analysis of Twitter data was undertaken to delineate the distribution of positive sentiments and their demographic correlates. Positive sentiments were categorized into clusters to streamline comparison across different age and gender demographics. The relationship between positive sentiment and vaccination uptake was evaluated using Spearman’s correlation coefficient. Additionally, a bivariate analysis was carried out to further probe public sentiment towards COVID-19 vaccines and their local adoption rates.

**Result:**

The results indicated that the majority of positive tweets were posted by males, although females expressed higher levels of positive sentiment. The age group over 40 dominated the positive tweets and exhibited the highest sentiment polarity. Additionally, vaccination uptake was positively correlated with the number of positive tweets and the age group at the local authority level.

**Conclusion:**

Overall, public opinions on COVID-19 vaccines are predominantly positive. The number of individuals receiving vaccinations at the local authority level is positively correlated with the prevalence of positive attitudes towards vaccines, particularly among the population aged over 40. These findings suggest that targeted efforts to increase vaccination uptake among younger populations, particularly males, are necessary to achieve widespread vaccination coverage.

## 1. Introduction

Since the onset of the COVID-19 pandemic in early 2020, the virus has spread globally, resulting in over 760 million confirmed cases and more than 6.8 million fatalities to date (1). Prior to the introduction of vaccines, the United Kingdom government employed non-pharmaceutical interventions (NPIs), such as national lockdowns and social distancing measures, in an attempt to mitigate viral transmission and prevent the National Health Service (NHS) from reaching capacity (2–4). A substantial body of literature attests to the efficacy of immunisations in preventing COVID-19 infections, indicating that vaccination is among the most reliable strategies for conferring long-term protection (5–8). In light of these findings, the United Kingdom government has issued guidelines promoting vaccination as a means of reducing the incidence of severe symptoms and mortality associated with COVID-19. Nevertheless, the effectiveness of such guidelines is predicated on the public’s willingness to comply and adhere to them [i.e., vaccination intention (9)] which may be influenced by their attitudes towards vaccinations. Consequently, it is imperative to examine public sentiment to gauge the receptiveness to the issued guidelines and evaluate the success of the vaccination campaign.

A conventional method for assessing vaccination intention involves conducting social surveys, which entail administering questionnaires to a specific group of individuals to collect data on their perspectives on vaccines. This approach excels in enabling researchers to obtain detailed and targeted information pertaining to the attitudes of a particular demographic. For instance, Paul et al. (10) employed weekly questionnaires to assess public attitudes towards the COVID-19 vaccine in the US, revealing that a majority of respondents harboured positive attitudes and were amenable to vaccination. However, this data collection method presents certain limitations. One potential drawback is the potential for bias stemming from non-random participant selection. If, for instance, the questionnaire is distributed exclusively to individuals with a pre-existing interest in vaccines, the results may not accurately represent the broader population (11). Another potential limitation is the social desirability bias, which refers to the propensity of respondents to report socially acceptable or desirable attitudes or behaviours rather than their genuine thoughts or actions (12). This bias can be particularly relevant when asking sensitive questions about controversial topics, such as vaccination. For example, Shaw et al. (13) reported that questionnaire respondents were more inclined to report favourable attitudes towards vaccines and less likely to disclose negative perspectives compared to those interviewed in person. This suggests that questionnaire-based methods may overestimate positive sentiments towards vaccines. Furthermore, administering questionnaires can be labor-intensive, time-consuming, and costly, which are common challenges associated with traditional data collection techniques. This is especially problematic when large sample sizes are required, as considerable time and resources may be expended in designing, administering, and analysing the questionnaire data.

Social media platforms, such as Weibo, Flikr, Twitter, and Facebook, have become increasingly important for assessing vaccination intention. For instance, Salathe and Khandelwal (14) analysed over 477,000 tweets to understand pulic sentiment towards the influenza A (H1N1) vaccine and found that social media data could be used to predict vaccination rates. Dunn et al. (15) conducted an anlsysis of exposure to information about HPV vaccines on Twitter and suggested that negative representations of vaccines in media, including safety concerns, misinformation, and conspiracy theories, may either reflect or influence vaccine acceptance. During the COVID-19 pandemic, social media platforms played a crucial role in understanding public sentiment towards the COVID-19 vaccines. Various studies worldwide were conducted to analyse the data and identify public concerns, misinformation, and acceptance of the vaccines (16–19). These examples imply that social media can be a valuable resource for evaluating vaccination intention and identifying potential obstacles to vaccine uptake since it offers a platform for individuals to voice their views and opinions publicly.

Utilising social media to investigate vaccination intention offers several advantages. Firstly, near real-time data collection enables rapid analysis and observation of changes in public sentiment over time, which is particularly valuable for understanding how attitudes may shift in response to events such as vaccine hesitancy or misinformation about vaccine safety (17). Secondly, geospatial attributes in social media data facilitate comprehensive spatial analysis, revealing spatial disparities in public sentiment towards vaccines (15, 20, 21). Lastly, social media grants researchers access to large and diverse samples, as a significant portion of the population uses these platforms. This high penetration is especially useful for analysing views of hard-to-reach individuals or understanding the sentiment of specific sociodemographic groups (22, 23).

However, it’s essential for researchers to consider inherent limitations such as potential bias due to self-selection of users and the challenge of accurately gauging sentiment from individual posts (24, 25). In line with our findings, In essence, Kobayashi et al. (26) demonstrated that Twitter data exhibits an age bias, predominantly featuring users over 40 years old. Furthermore, in sentiment analysis studies, there is a growing concern regarding different platforms manifesting distinct vaccination sentiments. Cesare et al. (27) suggested that despite the recent surge in social media usage, some demographic groups may remain under-represented on certain platforms. Conversely, Cascini et al. (28) found that various social media platforms tend to express comparable sentiments about COVID-19 vaccines concerning information polarisation. Additionally, Twitter has been highlighted as a primary platform for digital health monitoring and a valuable tool for opinion pieces during disease outbreaks (29–32) further pointed out the potential of social media for studying complex health-related information networks and providing cost-effective, real-time public health data. Notably, numerous studies [e.g. (16, 33–36),] have underscored the validity and significance of using social media data for analysing vaccine intentions.

Our study aims to undertake a sociodemographic and spatiotemporal sentiment analysis to unearth the spatial and temporal distribution of vaccine intentions by gender and age group, and critically examine the relationship between these intentions and actual vaccination uptake at the local authority level. While a wealth of studies have probed into the vaccine-related information disseminated on Twitter, a vast majority have not ventured into the exploration of the relationship between public sentiment towards vaccination and actual vaccine uptake. For example, Park and Suh (33) scrutinised public attitudes towards vaccination using Twitter data, but stopped short of examining the link between these attitudes and vaccination response. If sentiment analyses do not encompass real-world vaccination distributions, it could lead to a void in the utility of the findings, given the uncertainty surrounding the correlation between vaccination and emotional expression, thus limiting their contribution to policy making. Concurrently, while numerous studies have utilised Twitter data to delve into vaccine sentiment, their focus has predominantly been on topic modelling (26, 34, 36, 37) or sentiment categorisation (38). Such research often overlooks the important demographic characteristics, as well as spatial and temporal variables that could provide crucial insights for governments to fine-tune guidance to suit different regions or localities.

To address these gaps, our study presents a methodological framework for scrutinising COVID-19 vaccination intention and its uptake at the local authority level in England, focusing on real-world vaccination data and incorporating a wide array of critical variables. The framework consists of data extraction to capture vaccination data and Twitter data, data pre-processing to remove irrelevant content and tweets from institutional accounts recognised by M3, and subsequent spatiotemporal and sociodemographic sentiment analysis. The resulting insights are then used to investigate the relationship with vaccination uptake rates, contributing a more nuanced understanding of public sentiment towards COVID-19 vaccines and their actual use in the community.

This article is structured as follows: Section 2 delineates our methodological framework, explicating the process of data extraction and pre-processing applied to both Twitter and vaccination data. In Section 3, we present and dissect the findings of our sentiment classification. This section contains detailed subsections that explore the spatiotemporal distribution of different demographic characteristics of sentiment and analyse the relationship between sentiment and vaccination at two levels of granularity—the local authority and the national level, paying special attention to various demographic characteristics. The concluding section encapsulates our findings, enumerates the limitations of our study, and proposes avenues for future research in this domain.

## 2. Methodology framework

**Figure 1:**
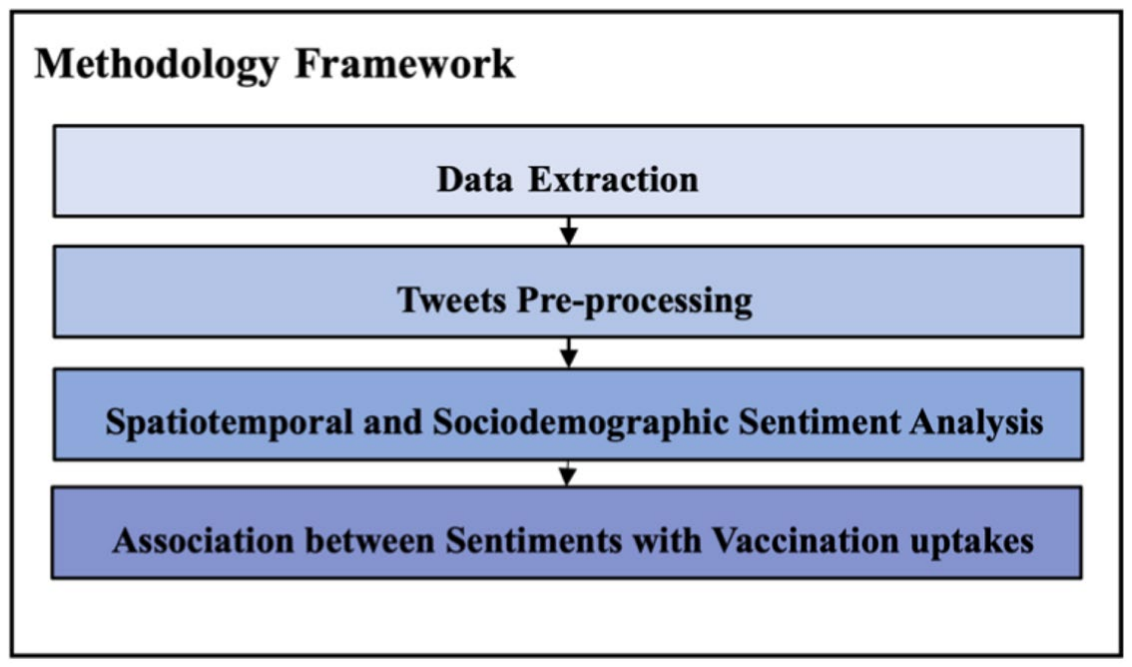
Methodology framework.

This research implements a comprehensive methodology framework, designed to visually depict the spatiotemporal distribution and demographic nuances of vaccination intention, and to quantify the correlation between vaccine intention and the actual vaccination rate. We perform spatiotemporal sentiment analyses by gender and age group, and explore the link between vaccination intention and vaccination rate, as shown in Figure 1, which includes 4 steps of data processing as follows:

1. Data extraction: this phase involves the extraction of vaccination and Twitter data. We utilised the SAIL Databank and the United Kingdom government API to capture demographic and spatio-temporal vaccination information, respectively. For Twitter data, we used TF-IDF to identify keywords related to vaccination from government documents, aiding in the targeted extraction of tweets.
2. Tweets pre-processing: in this step, we conducted data cleaning that included removal of retweets and advertisements. Following this, the M3 method was applied to filter out tweets from institutional accounts and classify the demographic characteristics of the remaining tweets.
3. Spatiotemporal and sociodemographic sentiment analysis: this part of the process employed VADER to categorize sentiments, followed by HDBSCAN to cluster areas with a concentration of positive sentiments.
4. Associations between sentiments and vaccination uptakes: we used Spearman’s correlation coefficient to examine the relationship between public sentiment and actual vaccination uptake. Spearman’s method was preferred over Pearson’s correlation as the sentiment scores, which typically range from −1 to 1, do not necessarily follow a normal distribution.

Our primary raw data includes vaccination data and Twitter data. The vaccination data is bifurcated into data from the Government API, offering the number of vaccinations in local authorities, and data from the SAIL database, which provides national-level vaccination figures along with sociodemographic information. Post extraction, Twitter data undergoes pre-processing and the cleaned data is utilised for spatiotemporal and sociodemographic sentiment analysis.

Sentiment analysis involves classifying users per sociodemographic characteristics using M3, clustering to discern temporal or spatial variances in users’ vaccination intentions, and sentiment polarity analysis coupled with the quantification of positive tweets. Typically, COVID-19 vaccine sentiment analysis follows a similar scheme, involving data pre-processing and sentiment categorisation by VADER (37, 39–41). However, the integration of M3 extraction for demographic characterisation with VADER for sentiment classification, while accounting for spatiotemporal factors, constitutes an innovative approach of our framework. This methodology results in outcomes of finer granularity and addresses the gap in existing research regarding the correlation between vaccines and sentiment.

In the final stage, the relationship between vaccine intention and uptake at the local authority level is examined using Spearman’s correlation coefficient. Subsequently, the interplay between these two variables is explored at the national level, focusing on socio-demographics.

### 2.1. Vaccination data

The study period, spanning from January 1, 2021, to April 1, 2022, incorporates various vaccination phases as advised by the United Kingdom government. These phases include the promotion of the first dose between January 2021 and April 2021, the second dose from April 2021 to October 2021, and the additional booster doses, recommended due to the emergence of the Omicron variant (42). The research period is thus divided into these three phases, each reflecting the roll-out of the corresponding doses.

The data for this study is derived from two primary sources: official United Kingdom government records and the SAIL Databank. Official Government Statistics, sourced from the British National Institute for Vaccination, were geographically aggregated based on the locations of the vaccinated individuals, then combined with the local authority shapefile for spatiotemporal analysis. The SAIL Databank data was profiled according to age and gender.

With significant population variation across different local authorities in England, data standardisation using population as the denominator is crucial for cross-regional comparability. The population dataset from official sources (43) is used for this purpose, and the spatial distribution of vaccinations at the local authority level is examined in relation to positive public sentiment.

To process the data from the SAIL Databank, an SQL query was used to extract information on dose sequences, age, gender, and local authority codes. The extracted data, combined with the local authority shapefile, helped focus on England’s vaccinated population. The number of vaccinated individuals was then categorised according to gender and age groups for further analysis. In the final dataset, only dose sequence, age, gender, and location code were retained for demographic screening.

**Figure 2:**
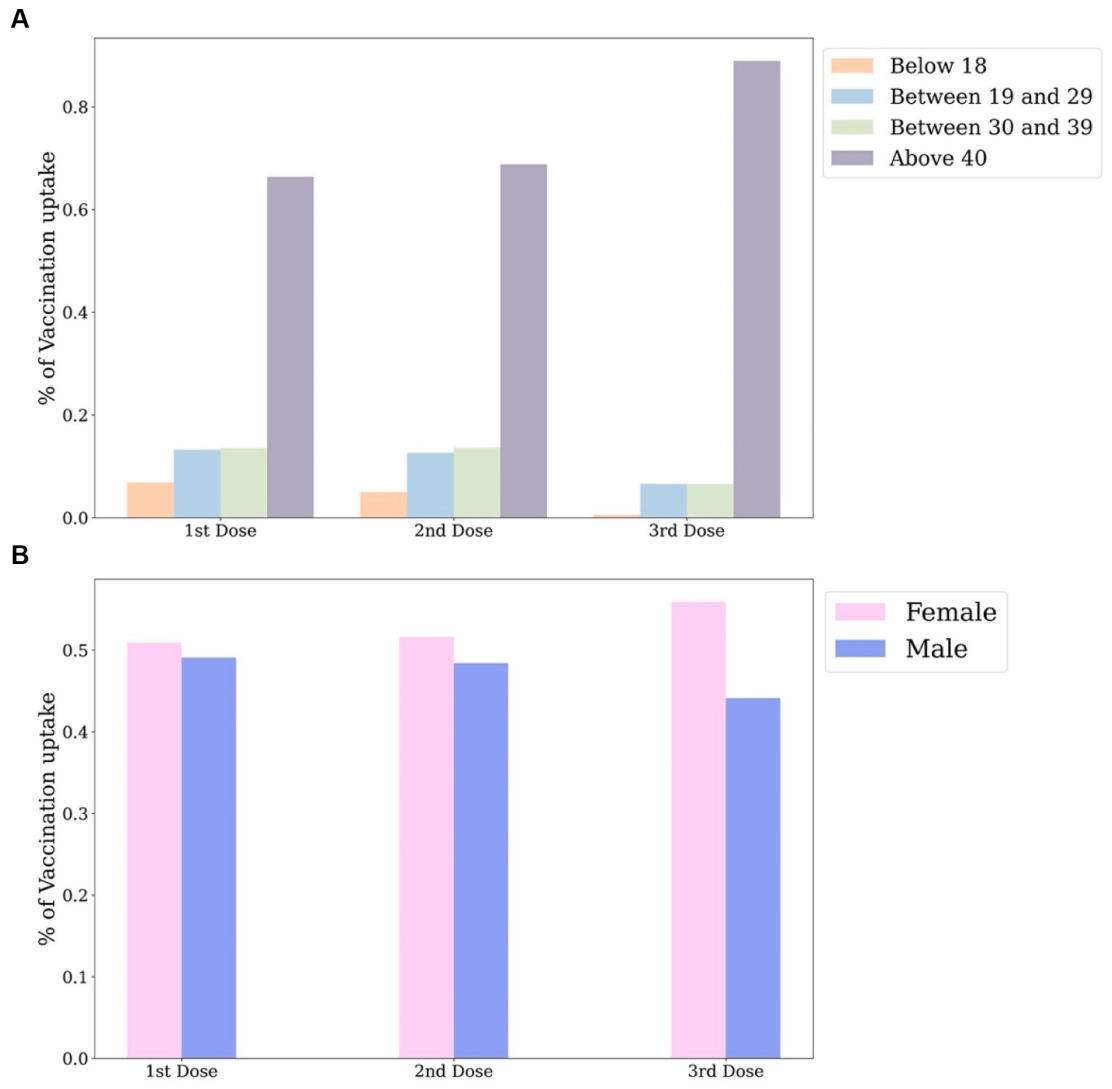
Distribution of positive tweets by age groups **(A)**, by gender **(B)** in national level.

A macroscopic view reveals that the distribution of the first two doses is relatively similar across all gender and age groups, as seen in Figure 2. This similarity is due to government guidelines that recommended two consecutive doses for maximum vaccine effectiveness. When analysing the age distribution of vaccine uptake in Figure 2A, it’s clear that individuals over 40 constitute the majority, with the number of vaccinations decreasing with age. The distribution by gender, shown in Figure 2B, indicates a higher number of vaccinated females than males, with the difference widening over the study period.

### 2.2. Twitter data extraction

In this study, Twitter, one of the most popular and widespread media platforms, was used to obtain vaccination intention through the use of academic API services and keyword searches. The COVID-19 vaccine guidelines were provided by the National Health Service^1^ and the Cabinet Office.^2^ Keywords related to vaccination were identified using the term frequency-inverse document frequency (TF-IDF) approach (44). Moreover, the names of approved vaccine manufacturers were extracted from a document supplied by the National Health Service (see footnote 1), which were also incorporated into the keywords for querying tweets. The results of the extractive summarisation are displayed in Table 1.

**Table 1:** The most pertinent word combinations for vaccination extracted by term frequency-inverse document frequency (TF-IDF) method for official government documents.

Keywords	Relevance	Keywords	Relevance	Keywords	Relevance
Vaccine	0.822	Moderna	0.075	Injection	0.035
COVID	0.448	Oxford	0.05	Vaccinated	0.035
Dose	0.224	Coronavirus	0.05	Immunization	0.035
Booster	0.149	Astrazeneca	0.05	Vaccination	0.025
Biontech	0.14	Valneva	0.035	Jab	0.025
Pfizer	0.1	Novavax	0.035	Janssen	0.025

Although not all terms exhibit a desirable high correlation in the extraction results, their low frequency of occurrence still renders them representative. Consequently, all phrases appearing in the table, along with the vaccine manufacturers’ names, were selected as keywords for tweet searches. Additionally, the language was set to English to facilitate comprehension of the expressed sentiment, retweets were filtered out, and advertisements were removed by setting “remove promoted content” to true in the query. These measures ensured the quality of the downloaded tweets, providing reliable and non-redundant information. As the raw data contained numerous unnecessary elements, such as conversation IDs, retweets, and like counts, data cleanup was necessary after geolocation filtering. To simplify the dataset, only the screen name, text, posting time and location, source, user name and ID, description, user profile URL, and coordinates were retained.

Further refinement excluded tweets originating outside of English local authorities and those from organisational accounts. To avoid any sentiment analysis bias, accounts with more than 50% abusive tweets were also eliminated. Redundant posts—defined as those submitted by the same user ID, in the same location, with more than five daily postings—were removed. Initially, the number of tweets collected were 50,994, 62,037, and 56,190 for the three phases, respectively. However, after data cleaning, these numbers reduced to 33,107, 37,401, and 22,494 tweets from 16,561, 18,387, and 11,874 unique users per phase.

Text preprocessing involved applying the tweet-preprocessor to clean up text, including URLs, mentions, reserved terms, emojis, and smileys. Subsequent steps included decapitalising words, removing spaces and punctuations, text normalisation (lemmatisation), and tokenising words in a phrase. The final step involved excluding stop words.

### 2.3. Estimating demographics—M3

Considering the potential bias in user characteristics across various social media platforms, results from studies focused solely on one platform might suffer from unrepresentative sampling. To mitigate analytical bias, it’s crucial to acquire adequate data on attributes like age demographics, sexual orientation, and organisational account status. Without factoring in these parameters, any analysis of tweets could lead to skewed results, either dominated by influential institutional accounts or by an overrepresentation of a particular age group or gender. To address such potential group bias, this study employs M3 inference recognition.

M3, a machine learning method for estimating demographics, was developed by Wang et al. (45) using a comprehensive Twitter dataset. The method is multimodal, multilingual, and multi-attribute, capable of recognizing textual and visual inputs, identifying 32 European languages, and concurrently predicting sociodemographic variables, including gender, individual or organisational account status, and users’ age groups. The M3 model’s limitations include considering only binary gender and achieving approximately a 40% precision in age classification. However, the overall Macro-F1 score reached 0.915 due to extensive data training. Its application is widespread in sentiment analysis using social media data, where it helps derive demographic information from such sources (46–48). Given such advantages provided by the M3 method, it was employed in this study to identify the social demographic characteristics and organisation status of Twitter users.

### 2.4. Sentiment analysis

Sentiment analysis, a prominent field in natural language processing, is often referred to as opinion mining. It aims to identify, extract, and organise attitudes from user-generated texts found on social media platforms, blogs, or consumer feedback (49). This technique is widely used to understand attitudes in human language text, assigning sentiment polarity scores to indicate positive, negative, or neutral sentiments. With the growing popularity of social media, sentiment analysis has become increasingly relevant in analysing the vast amount of subjective data available (50). In this study, the VADER (Valence Aware Dictionary and sEntiment Reasoner) technique will be employed to extract attitudes towards vaccination from social media text. Texts will be scored based on the probability of positive, negative, and neutral words, ultimately receiving a compound score for the overall sentiment. The compound score, ranging from −1 to 1, classifies pessimistic attitudes with scores below −0.05, optimistic perceptions above 0.05, and neutral sentiments in between.

One concern raised regards the fact that Twitter users are predominantly located in urban areas, meanwhile, as specified by the data on the government website (51) that areas with densely populated areas are predominantly metropolitan areas, which may bias the results. To mitigate the effect of population size on the number of tweets sent, data were divided by local authority population sizes provided by the United Kingdom government. Furthermore, in the three phases examined, approximately 51%, 47%, and 46% of total tweets exhibited positive attitudes, accounting for nearly half of the entire dataset. Consequently, only positive attitudes were carried forward for further analysis. Since the resulting outcomes seemed too insignificant to visualise differences, the average local tweets were summed as the denominator, allowing for a more comparable analysis across locations. However, considering the sentiment analysis results for each local authority in the United Kingdom, small amounts of positive sentiment in certain areas could lead to unfocused and unrepresentative results. Observations revealed that localities with higher volumes of positive sentiment were clustered in several groups of concentrated cities. By focusing on these localities, a relatively representative and aggregated distribution of positive tweets by gender and age can be obtained.

To gain deeper insight into the spatial distribution of positive attitudes towards vaccines, tweets with positive sentiments from each study phase were examined for clustering. Three clustering approaches were considered: DBSCAN, HDBSCAN, and multi-scale (OPTICS). HDBSCAN was chosen for this study due to its self-adjusting nature, eliminating the need to specify search distances for clusters (52). The algorithm automatically searches for optimal clusters by using varying distances; the only input required is the minimum cluster size, which determines the minimum number of points in each group. Aggregations with fewer points than the specified number are considered noise.

To examine the impact of parameters on clustering behaviour, the three separate study periods were subjected to HDBSCAN clustering. After several iterations, the minimum cluster size was set at 4% of the number of positive tweets within the given timeframe. This decision to define the minimum cluster size as a percentage of the total number of positive tweets per study period was driven by the inappropriateness of assigning an absolute value. The amount of data obtained for each study period varied, which could lead to non-comparable or biased results.

### 2.5. Relationship between sentiments and vaccine uptakes

The associations were analysed by examining the cumulative number of people vaccinated with different doses by date of vaccination and the counted number of positive attitudes posted online. Additionally, the percentage of vaccination uptake and sentiment polarities were paired for further analysis. Though Pearson’s product-moment correlation coeffieicnt could instruct the *p*-value of the correlation coefficient compared with the Spearman’s correlation coefficient, the assumption of Pearson’s product-moment correlation coefficient requires the data to be normally distributedIn the mean time, the sentiment polarities were scored ranging from −1 to 1, demonstrating the intensity of positive and negative emotions, the results were not normally distributed. Therefore, Spearman’s correlation coefficient, a quantitative method, was utlised. The Spearman correlation coefficient is a monotonic function which monotonically increases or either monotonically decreases, with singular relationship values varying from −1 to 1.

In order to assess the association between public attitudes towards vaccination and its uptake, Spearman’s correlation (53) was employed. The correlation coefficient was calculated using the number of positive attitudes posted pairwise in the related vaccinated population of different doses. It can be observed that there is a positive correlation between the number of favorable attitudes towards a vaccine and the number of respective vaccinated populations in the local authorities, indicating that an increasing number of encouraging attitudes expressed might promote the acceptance of vaccination.

## 3. Results

In general, the number of tweets sent by males was considerably higher than those posted by females concerning vaccines across all sentiments. Concurrently, the number of male users recognised by M3 is proportionately higher than the number of female users, whereas the gender distribution in England diverges from the situation, with more female residents than male residents. In addition, the non-binary population in England could not be taken into account due to technical limitations, whilst official population data from government websites have mentioned that over 93% of population reported same gender identity as registered at birth (54). However, most sentiment analyses on gender fail to fully account for the global non-binary population yet, as both males and females still predominate in numbers, which can be regarded as a robust representation, for example, Bathina et al. (46) conducted a gender sentiment analysis using social media data and drew solid conclusions. To address the issue of unequal gender use, a percentage was adopted to quantify positive attitudes towards vaccines across genders, i.e., the number of positive tweets across genders divided by the number of tweets sent across genders.

In terms of age distribution, M3’s results broadly align with the population distribution obtained from the United Kindom government website (55). However, there is a deviation in the youngest age group, which is dominated by the over-40s, with the proportion decreasing as age increases. To ensure result consistency, positive attitudes by percentage of age group have also been analyzed in the following sections.

The dataset for the second period is significantly larger due to different timeframes of the extracts. Throughout the reported survey period, there were 343, 191, and 116 vaccine-related tweets daily, indicating a decreasing interest in the controversial topic of vaccines over time.

In the first phase, positive attitudes towards vaccines dominated more than half of the sentiments, consistent with the findings of Lyu et al. (16) during a similar timeframe. However, in the next two phases, a decline in positive attitudes was observed, aligning with the research by Greyling and Rossouw (56).

Moreover, a positive relationship between vaccine intention and vaccination uptake was detected, highlighting the impact of encouraging messages online on promoting vaccination. However, vaccine intention declined over time, suggesting the need for further action to promote vaccine policy, which requires investigation into the influencing factors.

Our findings demonstrate the importance of investigating online sentiments towards vaccination to guide the government in targeted communication strategies based on emotions, demographics, and geographic areas. The detailed results will be discussed in the following sections.

### 3.1. Sentiment analysis

**Figure 3:**
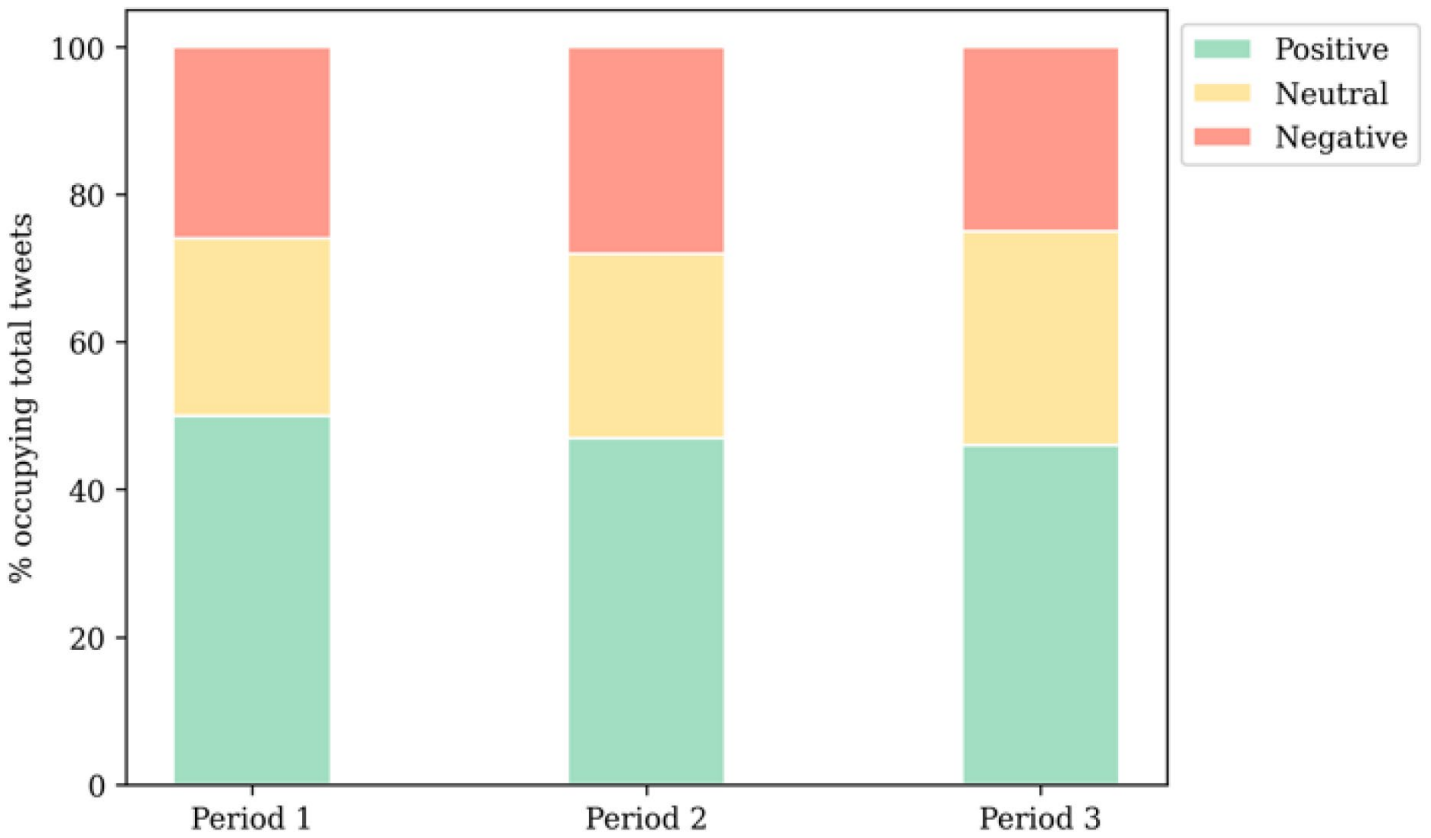
Distribution of sentiments among tweets.

Overall, as observed in Figure 3, during the first stage of vaccination, approximately half of the population expressed positive attitudes towards vaccination. However, the percentage of optimistic sentiments dropped to 47% and 46% in the subsequent two stages, though positive sentiments still constituted the majority. Despite the prevalence of optimism about vaccines throughout the study period, it remains uncertain whether the amount of optimism positively correlated with its intensity. In other words, the possibility of few but strong negative attitudes coexisting with numerous but generally neutral positive ones requires further investigation. Consequently, the mean value of positive sentiment polarity was calculated to examine the average intensity of individuals’ attitudes towards vaccines. Encouragingly, the average sentiment polarity for each study phase was approximately 0.58, 0.57, and 0.55, respectively. While a decrease in polarity over time can be observed, these values were significantly higher than the neutrality threshold, indicating a robust and predominantly positive attitude towards vaccines, aligned with the corresponding survey from Yin et al. (37) that the overall sentiment polarity is positive. Hence, the spatio-temporal analysis of public sentiment towards vaccines focused only on tweets with positive attitudes.

**Figure 4:**
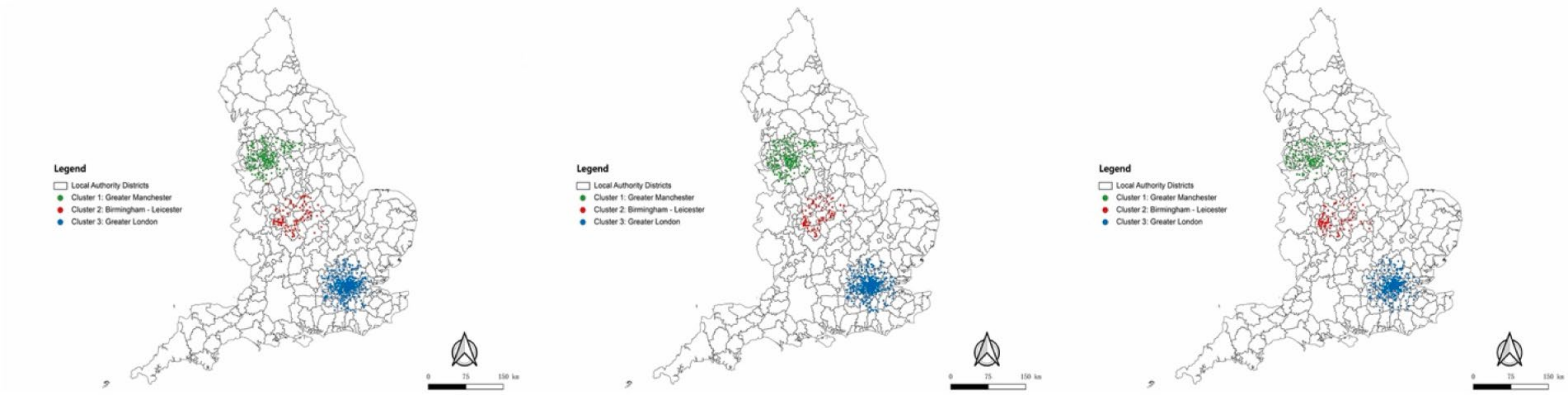
Clusters of positive attitudes over time by using HDBSCAN: left graph for the first phase, middle graph for the second phase, right graph for the third phase.

As seen in Figure 4 for the three periods, the overall clusters were similar among these periods around Greater Manchester, Birmingham and Leicester, and Greater London and its neighbours, indicating that the areas with positive attitudes remained consistent throughout the investigation period. Since these clusters are representative, this section’s analysis focused on these specific groups. To facilitate further discussion on the spatiotemporal analysis, the areas surrounding Greater Manchester, Birmingham and Leicester, and Greater London and its neighbours, as illustrated in the graph above, were labelled as GM, BL, and GL, respectively, in the subsequent parts of the analysis.

### 3.2. Sentiments vs. demographics

**Figure 5:**
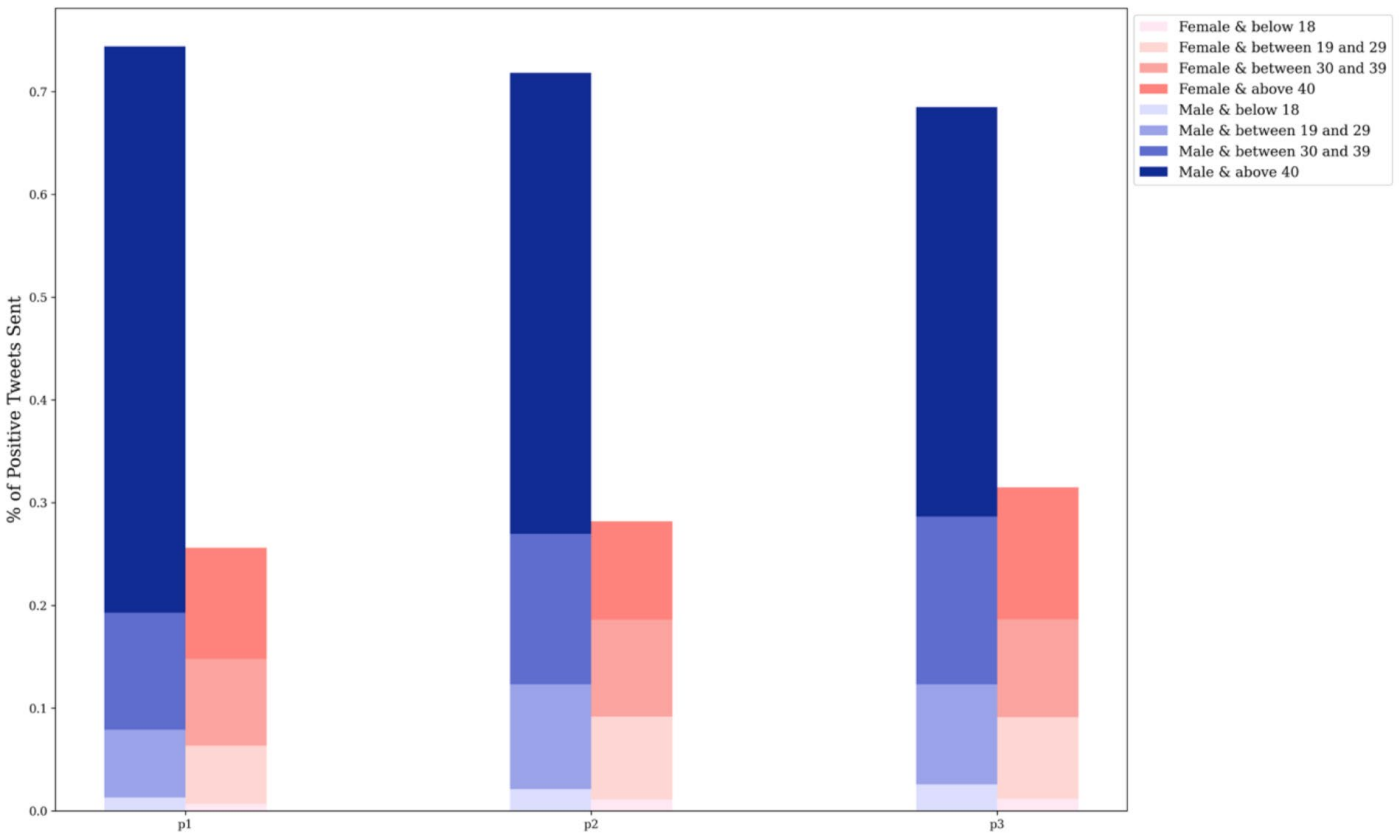
Distribution of positive tweets by gender and age group.

Figure 5 presents the distribution of positive tweets concerning vaccination posted by different groups, taking into account differences in gender and age throughout the entire investigation period. It is noteworthy that the volume of positive tweets posted by males is considerably larger than those posted by females. Furthermore, within both the male and female groups, users aged over 40 were found to dominate the number of positive vaccine-related tweets sent.

**Figure 6:**
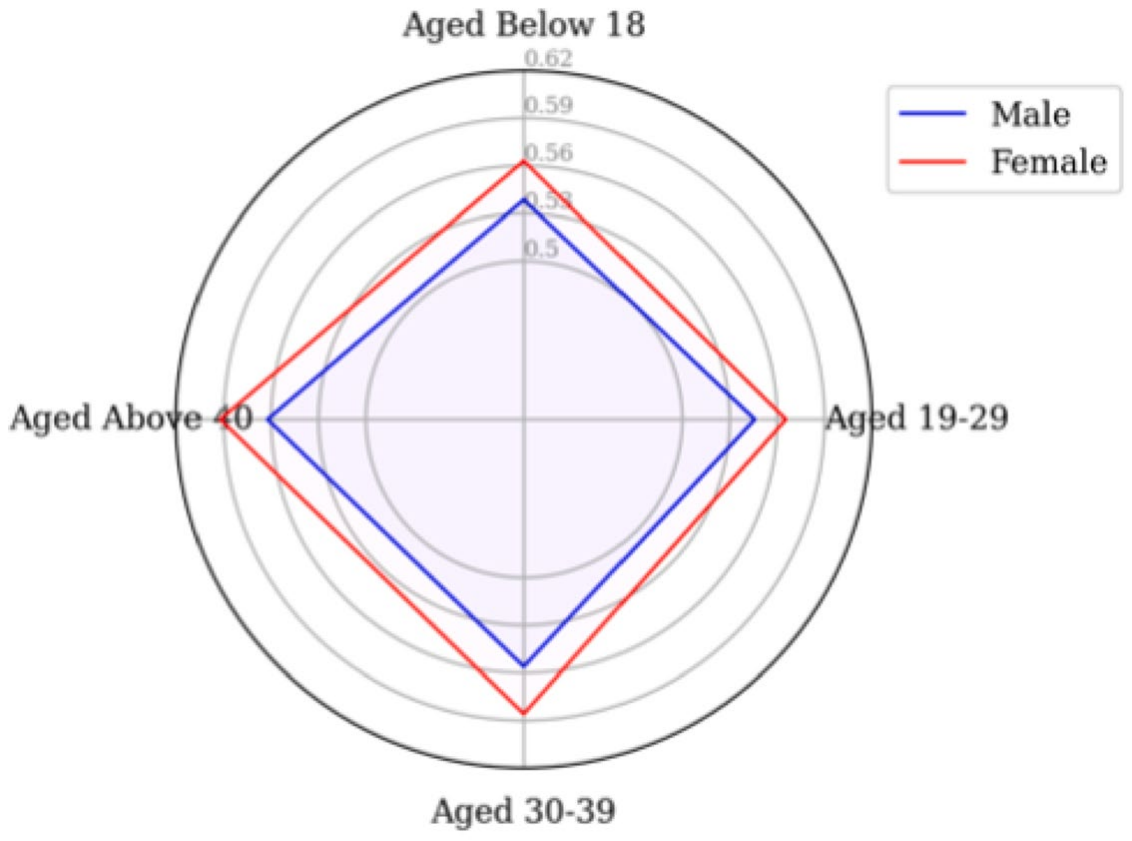
Sentiment polarities by gender and age group.

As previously mentioned, examining the number of tweets alone is insufficient to provide a comprehensive understanding of public sentiment; emotional polarities must also be considered. Figure 6 reveals a surprising finding: although males posted a significantly higher number of positive tweets compared to females, the sentiment polarities were higher for females across all age groups. This suggests that while male users may have been more active in discussing vaccines, female users expressed stronger optimism about vaccines.

#### 3.2.1. Spatio-temporla change of sentiments with gender

**Figure 7:**
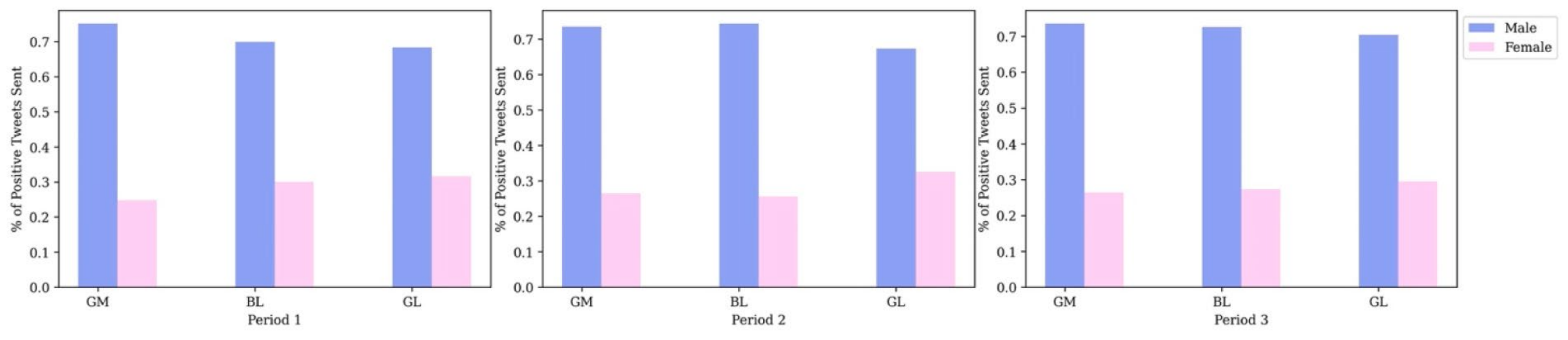
Percentage of positive attitudes over time of males and females by clusters.

In general, Figure 7 demonstrates that male users expressed considerably more positive sentiments than female users. Initially, female users posted far fewer positive tweets about vaccination compared to male users. However, the gap between the two groups diminished over time. This indicates that the rate of growth in the number of vaccine-related positive tweets posted by females relative to male users increased more rapidly during the second and third study periods.

**Figure 8:**
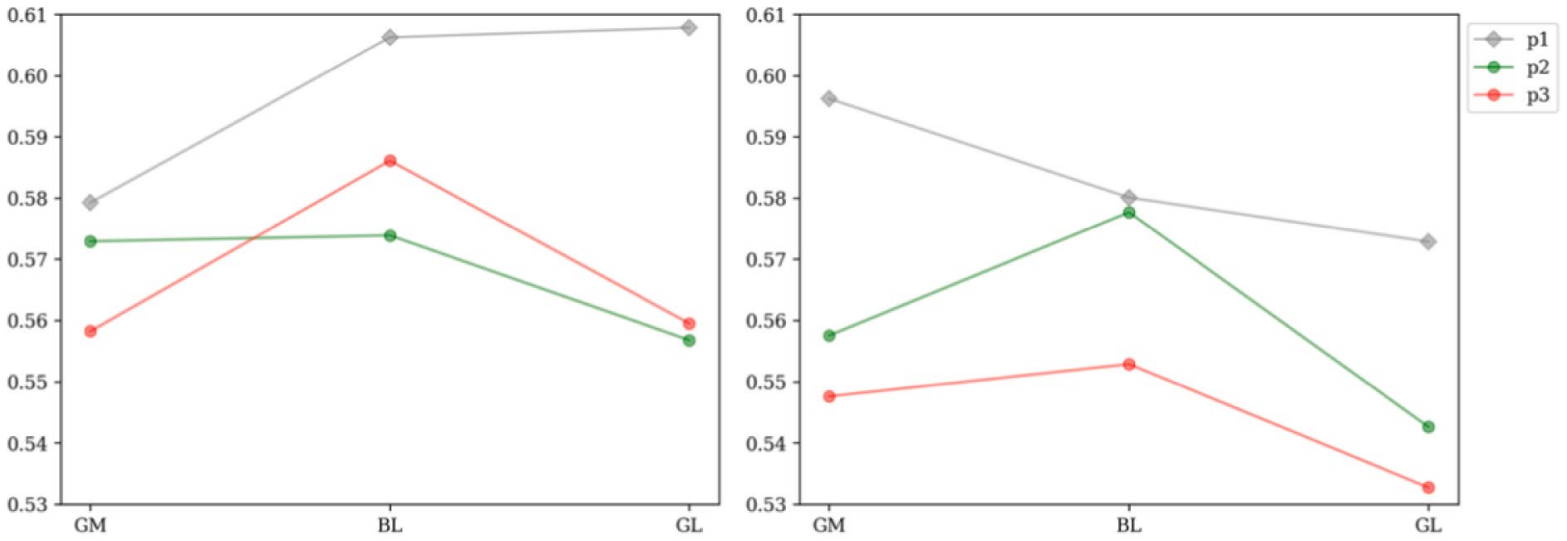
Sentiment polarities over time by gender and clusters: female positive sentiment polarity (left), male positive sentiment polarity (right).

In Figure 8, focusing on the GM cluster, it is evident that during phase 1, the emotional polarity expressed by males remained higher than that of females. However, in phase 2, females’ polarity surpassed males’ and maintained a comparatively high level in phase 3. For subgroup three, females exhibited higher affective extremes than males across all three periods, with an outstanding finding that in the first period of this cluster, females’ affective intensity was comparably high to any other group in any period and significantly higher than all males’ sentiment polarities in the same interval. This group, similar to the BL cluster among females, scored the lowest of all temporal segments in period 2. Overall, the positive attitudes expressed by male users decreased over time, as the grey line consistently remained above the other two lines, while the pink line was consistently at the bottom. Spatially, both male and female emotional polarities in the BL cluster attained greater prominence than those in the GM and GL clusters during the study period when the second dose and booster were introduced, aligning with the overall spatial-temporal findings. Generally, across different temporal and spatial analyses, males exhibited no sentiment polarity scores above 0.6, while females showed none below 0.55, which supports the observation that female sentiment polarities were generally higher than those of males in the sentiment-age analysis.

#### 3.2.2. Spatio-temporla change of sentiments with age

**Figure 9:**
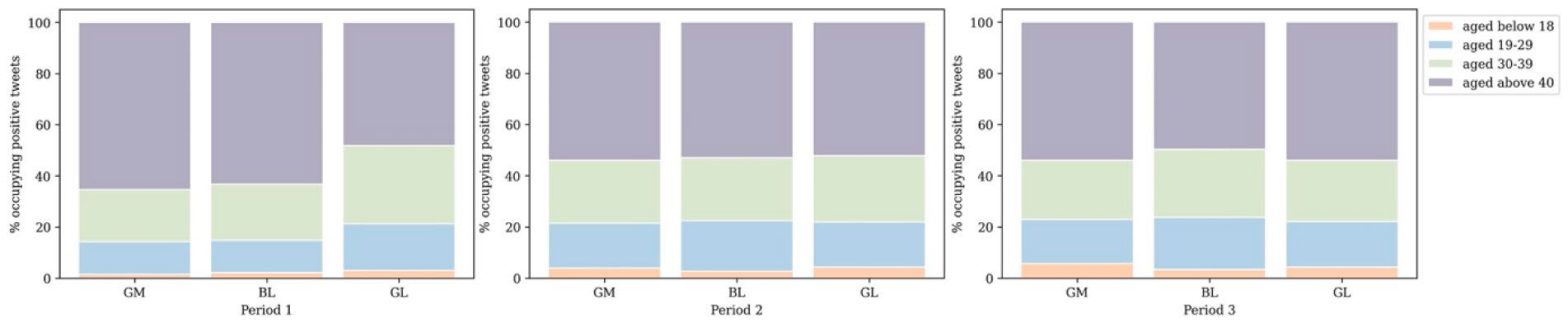
The percentage of positive attitudes over time by age groups and clusters.

**Figure 10:**
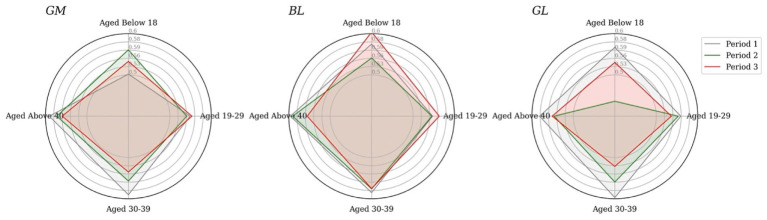
Sentiment polarities over time by age group for cluster1 (left), cluster 2 (middle), cluster 3 (right).

Considering the volume of positive attitutdes towards vaccine in Figure 9, spatially, it becomes apparent that the age structures of the three clusters were consistent throughout the study duration, with the majority being over 40 years old and the minority being under 18 years old. As for sentiment polarities in Figure 10, regarding age-specific emotional intensity, those aged 40^+^ consistently exhibited the highest scores in GM compared to other age groups, even with a decline in positive attitudes towards vaccines over time, aligning with the proportion of positive tweets sent. In contrast, the distribution of emotional polarity in BL and GL did not correspond with the number of positive tweets. Notably, in GM, the under-18 age group demonstrated the highest level of confidence in vaccines at Stage 2, while in BL and GL, the lowest level of optimism at Stage 2 was evident in the youngest group of Twitter users, who experienced the most fluctuations in sentiment.

A potential explanation for the low number of positive tweets and fluctuating emotional polarity among the under-18 group is insufficient knowledge about vaccines. Across all age groups, a downward trend in positive sentiment among 19 to 29 years-olds is apparent, which does not align with the share of positive tweets. Compared to the other clusters, BL presents the largest quadrilateral area over the three study periods, suggesting consistently higher sentiment poles than the other two groups, corroborating the results of the spatio-temporal analysis.

### 3.3. Association between sentiments with vaccination uptakes

Table 2 shows the correlation coefficient of the vaccination and attitudes towards vaccines in the three vaccination phases, indicating a positive correlation between the number of vaccination injections and the number of affirmative tweets.

**Table 2:** Spearman’s correlation coefficient results for vaccines and corresponding period of public attitudes volume.

	Number of first dose uptake	Number of second dose uptake	Number of third dose uptake
Correlation coefficient	0.54	0.54	0.53

Compared to the correlation between the population and the number of affirmative attitudes, the relationship between sentiment polarities and the vaccination rate *per capita* in local authorities (Table 3) appears smaller. This illustrates that the positivity intensity conveyed online does not significantly encourage increased willingness to receive vaccines, and hence, it will not be further discussed in this study.

**Table 3:** Spearman’s correlation coefficient results for vaccines and corresponding period of sentiment polarities.

	1st dose *per capita*	2nd dose *per capita*	3rd dose *per capita*
Correlation coefficient	0.13	0.09	0.16

**Figure 11:**
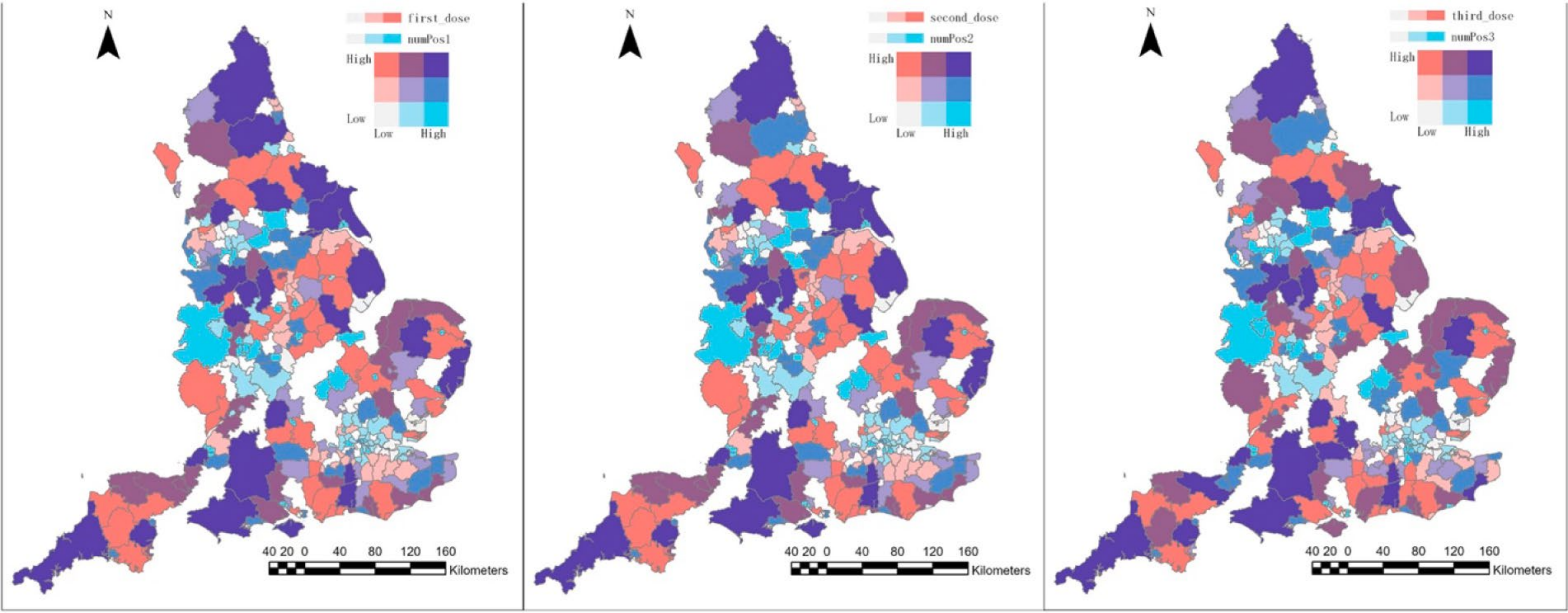
Bivariate graph of positive tweets distribution and vaccination uptake distribution in local authiorites level for first dose (left), second dose (middle), third dose (right).

Additionally, observing Figure 11, there appears to be a roughly identical pattern for the first two periods, which aligns with the government policy that subsequent two doses uptake can maximise the effectiveness of the vaccination. Throughout the entire investigated period, only small variations in Figure 11 indicate that individuals’ online perceptions of vaccines remained consistent during the study period in terms of their exposure to realistic practices.

Meanwhile, although activism exerted one of the dominant groups in London, the vaccinated population was not as well-represented as expected, nor was the northwest of England. In the case of London, a possible explanation for the significant variation between the number of positive attitudes and the number of people vaccinated could be attributed to considerable population movements in London and its vicinities. For instance, people visiting London for tourism or work purposes may have delivered positive tweets related to vaccines during this period without receiving vaccinations in London, which drove an increase in the number of positive tweets accompanied by a lower vaccination rate locally.

However, it is essential to note that the number of positive tweets is probably not the only factor accounting for the low vaccination rates in these areas. Additional factors, such as socio-economic factors, should be taken into consideration in a linear regression analysis for further investigation.

### 3.4. Age and gender of vaccination and positive attitudes

**Figure 12:**
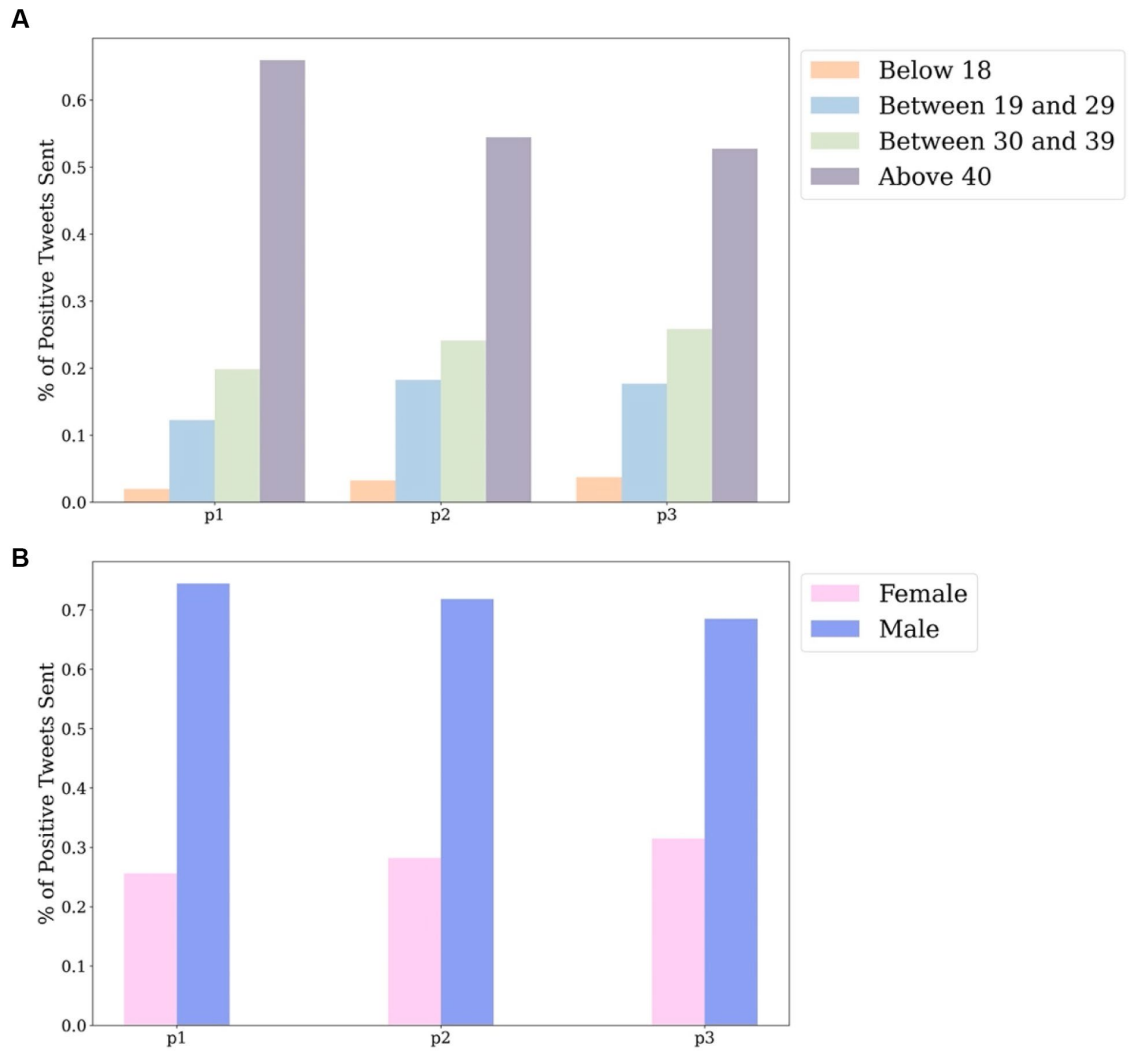
Distribution of positive tweets by age groups **(A)**, by gender **(B)** in national level.

Based on the age distribution of the vaccination population depicted in Figure 2A, it is evident that the majority of individuals who received the vaccine were older than 40 years, whereas the lowest number of recipients belonged to the age group younger than 18 years. This finding is consistent with Figure 12A, which displays the lowest number of proactive responses related to vaccines in the age group under 18 years, thus supporting the hypothesis of insufficient knowledge about vaccines in this age group. To address this issue, the government could enhance public awareness of vaccines among young people by promoting the advantages of vaccine uptake and encouraging parental confidence in the vaccine.

From a gender-based perspective of the vaccination population, it can be ascertained that women are more receptive to the vaccine than men in all three doses (Figure 2B). Conversely, the number of constructive attitudes is about double the number of positivity declared by men in relation to vaccination than that conveyed by women at the national level (Figure 12B). Consequently, it is inferred from the variation in the results of vaccination coverage and positive tweets in the gender distribution that categorising individuals by gender to predict their future behavior may not be a well-informed reference and, in reality, could result in bias.

## 4. Conclusions and further work

### 4.1. Findings

This study aimed to investigate public sentiment towards vaccination by analysing sentiment on Twitter in local authorities level and considered demographic characteristics across England. Additionally, it sought to identify the distribution of vaccinated populations and investigate the relationship between vaccines and public attitudes. The data collection period was from January 1, 2021 to April 1, 2022, and it was split into three phases in accordance with the national vaccination scheme’s roll-out and peak volume of vaccinations at each dose.

Generally, the sentiments towards vaccine remained neutral and positive occupying over 70% of total sentiments, while sentiment polairties remained over 0.5, congruent findings with Canaparo et al. (34). The results suggest that the number of positive attitudes towards vaccines is dominated by males and the age group above 40, while females express more positive sentiment polarities towards vaccines. From a spatial perspective, GM, BL and GL were identified as clusters with the most positive attitudes towards vaccines. Temporally, both the volume of tweets endorsing the vaccine and sentiment polarities declined throughout the study period, indicating a potential increase in vaccine hesitancy.

The distribution of vaccination numbers for the first two doses followed a similar pattern, aligning with the government’s recommendation that two consecutive doses would improve vaccine effectiveness. However, there was a sharp decrease in the number of booster injections. It is possible that there was a deficiency in valid promotion from a governmental perspective or hesitancy and a deterioration in trust in the vaccine from an individual standpoint.

The correlation analysis showed a positive association between the number of vaccinations and positive attitudes expressed on social media. However, noticeable differences were observed in certain geographical areas, such as London and its neighbours, which require further investigation. Gender-based analysis revealed a significant difference between the proportion of positive messages posted and the corresponding proportion of vaccinations, suggesting that gender may not be a reliable predictor of individual behaviour. On the other hand, age group analysis showed that the vaccination rates corresponded to the number of positive attitudes, with the 40^+^ age group dominating positive emotions. This suggests that age group classification may be a more reliable predictor of future behaviour based on social media sentiment analysis.

In summary, during the first phase (March 11, 2020–January 31, 2021), positive attitudes towards vaccines accounted for more than half of the sentiments. However, in the subsequent two phases, there was a noticeable decline in positive attitudes, indicating the need for further action to promote vaccine policy. These findings align with existing research in this area.

Additionally, our research has uncovered three key new findings:

- Female sentiment polarities were generally higher than those of males in the sentiment-age analysis.
- The young age group showed a low number of positive tweets and fluctuating emotional polarity.
- A positive correlation was observed between vaccination intentions and actual vaccination rates.

These discoveries provide crucial insights for governmental bodies. Furthermore, the methods and frameworks used in this study can be adapted to other countries or regions. The findings from the case studies conducted in United Kingdom big cities have the potential to assist governments worldwide in devising targeted strategies for different regions or age groups, thereby enhancing policy promotion programs and improving public health outcomes. For instance, combining the spatio-temporal aspect with the gender and age group perspective could figure out solutions to specific groups with different locations temporally.

### 4.2. Limitations and further research

This study has certain limitations that should be taken into account. Firstly, it focused exclusively on examining the relationship between vaccination volume and perceived attitudes towards vaccines, without delving into a comprehensive analysis of the factors contributing to the discrepancy between vaccination volume and vaccination intention.

Furthermore, it is crucial to acknowledge the inherent biases in datasets collected from Twitter. For example, Kobayashi et al. (26) reported biased data from specific age groups. Although all datasets possess inherent biases, Twitter datasets remain accessible, cost-effective, and offer extensive coverage, making them conducive to sentiment analysis in surveys.

Additionally, the gender classification method (M3) used in this study considered only binary populations, disregarding approximately 6 percent of the population. While this may constitute a small minority, it is essential to recognise and address inclusivity considerations in research. Despite the majority binary population in the United Kingdom contributing to the credibility of our findings, it is advisable to exercise caution in applying these conclusions in certain contexts.

Future research should strive to identify any spatial patterns in vaccination distribution and the distribution of positive sentiment. Incorporating socio-economic factors, such as income, education, and professions, into the regression model could aid in developing an optimised model for this purpose. If spatial patterns are detected, employing a spatial regression model can help reveal the factors that spatially influence vaccination.

The findings of future research hold the potential to identify pathways for improving vaccination efforts and promoting positive attitudes towards vaccines, providing valuable insights for authorities seeking to enhance public health initiatives.

## Data availability statement

The original contributions presented in the study are included in the article/supplementary material, further inquiries can be directed to the corresponding author.

## Author contributions

TC and BH: conceptualisation and methodology. BH: software, validation, resources, and writing-original draft. BH and YL: investigation, data curation, and visualisation. TC, BH, and YL: writing-review and editing. TC and YL: supervision. TC: project administration and funding acquisition. All authors contributed to the article and approved the submitted version.

## Funding

The research is supported by the MRC Grant Ref: MC PC 19,070 awarded to UCL on 30 March 2020 and MRC Grant Ref: MR/V028375/1 awarded on 17 August 2020.

## Conflict of interest

The authors declare that the research was conducted in the absence of any commercial or financial relationships that could be construed as a potential conflict of interest.

## Publisher’s note

All claims expressed in this article are solely those of the authors and do not necessarily represent those of their affiliated organizations, or those of the publisher, the editors and the reviewers. Any product that may be evaluated in this article, or claim that may be made by its manufacturer, is not guaranteed or endorsed by the publisher.

## Footnotes

^1^https://www.nhs.uk/conditions/coronavirus-covid-19/coronavirus-vaccination/coronavirus-vaccine/ (Accessed April 14, 2022).

^2^https://www.gov.uk/government/publications/covid-19-response-living-with-covid-19/covid-19-response-living-with-covid-19 (Accessed April 14, 2022).
